# Determination of Optimal Parameters for Dual-Layer Cathode of Polymer Electrolyte Fuel Cell Using Computational Intelligence-Aided Design

**DOI:** 10.1371/journal.pone.0114223

**Published:** 2014-12-09

**Authors:** Yi Chen, Weina Huang, Bei Peng

**Affiliations:** 1 School of Mechatronics Engineering, University of Electronic Science and Technology of China, Chengdu, 611731, China; 2 Robotic Research Center, University of Electronic Science and Technology of China, Chengdu, 611731, China; 3 School of Engineering and Built Environment, Glasgow Caledonian University, Glasgow, G4 0BA, United Kingdom; North China Electric Power University, China

## Abstract

Because of the demands for sustainable and renewable energy, fuel cells have become increasingly popular, particularly the polymer electrolyte fuel cell (PEFC). Among the various components, the cathode plays a key role in the operation of a PEFC. In this study, a quantitative dual-layer cathode model was proposed for determining the optimal parameters that minimize the over-potential difference 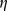 and improve the efficiency using a newly developed bat swarm algorithm with a variable population embedded in the computational intelligence-aided design. The simulation results were in agreement with previously reported results, suggesting that the proposed technique has potential applications for automating and optimizing the design of PEFCs.

## Introduction

As a result of the increasing need for an efficient and clean energy supply, considerable importance has been placed on the advancement and fundamental research of polymer electrolyte fuel cell (PEFC) technology. Among the components of PEFCs, the cathode plays a key role in the operation of PEFCs, in which an oxygen reduction reaction (ORR) occurs and generates heat. Platinum (Pt) loading, ionic conductivity, and the reaction's exchange current density are among the factors that may affect the performance. Numerous studies have been conducted to develop models and approaches that are essential to battery performance and optimization.

Springer et al. [1] presented an isothermal, one-dimensional, steady-state model for a PEFC with a Nafion 117 membrane, in which the water diffusion coefficients, electro-osmotic drag coefficients, water sorption isotherms, and membrane conductivities were employed. Bernardi and Verbrugge [2] developed a mathematical model of the solid-polymer-electrolyte fuel cell, and they utilized this model to investigate the factors that affect the performance of the fuel cell and to elucidate the mechanism for the transport of species in a complex network of gas, liquid and solid phases. Amphlett et al. [3] reported a parametric model for predicting the performance of a solid PEFC by considering the mass transport properties. Bevers et al. [4] presented a one-dimensional dynamic model of a gas diffusion electrode that considered the various effects of parameter changes. Kulikovsky [5] developed a two-dimensional model of the cathode compartment of a PEFC with gas channels. Rowe and Li [7] proposed a one-dimensional non-isothermal model of a PEFC to investigate the effects of various design and operating conditions on the cell performance. Baschuk and Li [6] formulated a mathematical model for the performance and operation of a single PEFC. Song et al. [8] utilized the AC impedance method to optimize the thickness and composition of the supporting PEFC layer. Ramadass et al. [9] developed a semi-empirical approach of capacity fade prediction for Li-ion cells, which considers the active material and rate capability losses. Wang et al. [10] investigated the effects of different operating parameters on the performance of PEFCs through an experiment that employed pure hydrogen on the anode side and air on the cathode side. Yerramalla et al. [11] developed a mathematical model to investigate the dynamic performance of a PEFC with a number of single cells combined into a fuel cell stack. Song et al. [12] investigated one- and two-parameter numerical optimization analyses of PEFC cathode catalyst layers that consider the Nafion content, Pt loading, catalyst layer thickness and porosity. Grujicic and Chittajallu [13] developed a model for determining the air-inlet pressure, cathode thickness and length, and the width of the shoulders in the inter-digitized air distributor. Weber and Newman [14] reviewed the models of PEFCs, the general modeling methodologies and some related summaries. Pathapati et al. [15] reported a mathematical model for simulating the transient phenomena in a PEFC that can predict the transient response of cell voltage, temperature, hydrogen/oxygen out flow rates and cathode and anode channel temperatures/pressures under sudden changes in load current. Tao et al. [16], [17] developed a three-dimensional, two-phase and non-isothermal model and carried out the parameter sensitivity analysis. Wang et al. [18] utilized a three-dimensional model to analyze the effect of the design parameters in a bipolar plates with the serpentine flow field. Wang and Feng [19]–[21] reported a one-dimensional study on electrochemical phenomena within the cathode. Wang et al. [22] reviewed recent PEFC technical progress and applications, the role of fundamental research in fuel-cell technology and the major challenges in fuel-cell commercialization. Khajeh-Hosseini-Dalasm et al. [23] proposed a computational study of the cathode catalyst layer of a PEFC and structural parameters analysis. Gao et al. [24] presented a multi-physical dynamic fuel cell stack model. Wang et al. [25], [26] investigated an inverse geometry design problem for optimization of single serpentine and transient characteristics of PEFC with parallel and interdigitated flow fields using a three-dimensional, two-phase model. Jung et al. [27] developed an elaborate simulation model of the fuel cell stack system. Askarzadeh and Rezazadeh [28] proposed an innovative global harmony search algorithm for parameter identification of a SR-12 Modular polymer electrode membrane(PEM) Generator. Wang et al. [29] carried out the parameter sensitivity analysis for a three-dimensional, two-phase, non-isothermal model of polymer electrolyte membrane fuel cell. Chen et al. [30] proposed a quantitative approach for predicting the remaining battery life by using an adaptive, bathtub-shaped function. Considering thermoelectric and thermoeconomic objectives, Sayyaadi and Esmaeilzadeh [31] developed a methodology for optimal PEFC control, in which the net power density and energetic efficiency are maximized. Pathak and Basu [32] discussed a mathematical model for the anode and cathode with an anion-exchange membrane for predicting the performance of a fuel cell considering reaction kinetics and ohmic resistance effects. Noorkami et al. [33] investigated the temperature uncertainty as a key parameter in determining the performance and durability of a PEFC. Molaeimanesh and Akbari [34] proposed a three-dimensional lattice Boltzmann model of a PEFC cathode, in which the electrochemical reaction on the catalyst layer is able to simulate single- and multi-species reactive flow in a heterogeneous, anisotropic gas diffusion layer. Wang et al. [35] studied a three-dimensional, two-phase, and non-isothermal fuel cell model incorporating the Leverett-Udell correlation and evaluated its performance.

Although there have been a large number of previous studies, the available literature on the analytical modeling of cathode electrodes fails to address two concerns. First, the previous studies do not capture the coupling effects on PEFC performance resulting from the interactions among the design variables. Second, few effective methods have been developed that allow for quantitative analysis, model verification, and parameter optimization. To fill this void, this paper proposes a bat swarm algorithm with a variable population (BAVP) to construct and optimize the quantitative cathode electrode model, which will be embedded into the computational intelligence-aided design (CIAD) [36] framework. This new CIAD framework provides an expanded capability to accommodate a variety of CI algorithms, and it has three advantages: (1) mobilizing computational resources; (2) taking advantage of multiple CI algorithms; and (3) reducing computational costs. This framework has been demonstrated in some of our previous works in diverse areas: applied energy [30], new drug development for public healthcare [37], [38], economy and finance [39], sustainable development [40]–[42], aerospace engineering [43], automotive engineering [44], public security [45], and engineering modeling and design [46], [47], among others.

Inspired by the echolocation behavior of bats and first proposed by Yang [48] in 2010, the bat swarm algorithm (BA) allocates computational resources by adjusting its population and accelerating the calculation speed. By using echolocation, a swarming bat can quickly respond to changes in the direction and speed of its neighbors during activities such as detecting prey, avoiding obstacles, and locating roosting crevices in dark surroundings. Useful behavioral information is passed among bats and guides them to move from one configuration to another as one unit. By borrowing this intelligence of social behavior, the BAVP is parallel, independent of initial values, and able to achieve a global optimum.

This work has three main contributions. First, our model can effectively assess PEFC performance. Second, a BAVP swarm intelligence method is devised as the search engine to optimize the model parameters embedded in the CIAD framework. Third, two new metrics, the index of moving mean of the average precision (mmAP) and the index of moving mean of variance (mmVAR), are introduced to characterize the dynamic evolutionary behaviors.

The remainder of this paper is organized as follow: Section 2 (Analytical Modeling) discusses the analytical modeling of the cathode electrode; Section 4 (Computational intelligence-aided design) describes the conceptual framework of CIAD and the integrated solver; Section 3 (Bat swarm algorithm with variable population) describes the BAVP algorithm for the optimization; Section 5 (Optimization and Parameter Determination) defines the fitness function for optimizing the analytical model using the model proposed in Section 2; Section 6 (Empirical Results and Discussion) presents the empirical results and further verifies the optimal design; and Section 7 (Conclusions and Future Works) concludes the paper.

## Analytical Modeling

**Figure 1:**
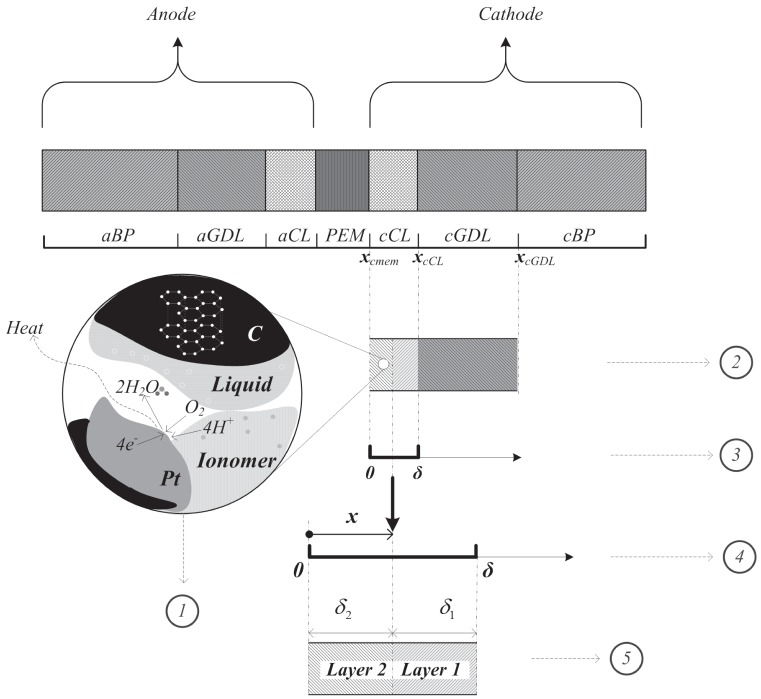
Schematic of a dual-layer cathode electrode of a PEFC.

A schematic diagram of a dual-layer configuration of a cathode electrode is shown in Fig. 1, in which five specific areas are numbered and are explained below. The left side of the electrode attaches to the PEM, and the right side connects to the diffusion media [19], [20].

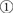
 Includes the assumption that the oxygen concentrations, temperatures, electronic phase potentials, and equilibrium potential are the same between the two layers and are uniform within each layer. The electrodes are thin layers (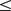
10
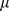
m) coated on the PEM surface containing a catalyst (typically **Pt**), carbon(**C**), an ionomer electrolyte and void space. In general, there are three phases in the electrode: (i) void space for the transport of gaseous reactants, (ii) ionomer content for the transfer of protons, and (iii) carbon support for conducting electrical current. In addition to the electrochemical catalyst, which is essential for all functions, equation (1) is given.
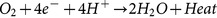
(1)

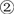
 The two sub-layers are denoted as ‘Layer 1’ and ‘Layer 2’. Five parameters are considered in this model that include the ionic conductivity 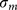, the catalyst specific area 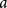, the exchange current density 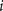, the ionic resistance 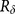, the current density 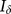, the thickness of sub-layer 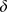, the interface location of the two sub-layers 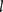, and so on.

The ionic conductivity factors 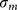 of ‘Layer 1’ and ‘Layer 2’ are 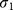 and 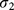, respectively, and they are determined from the electrolyte water content 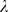, the ionomer tortuosity 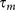, the Nafion content 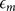 and the temperature 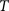, as given in equation (2). The ratio of the ionic conductivity factors of ‘Layer 1’ and ‘Layer 2’ is given in equation (3).
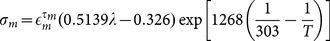
(2)

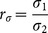
(3)


The catalyst specific area 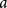 describes the active catalyst surface area per unit volume. The exchange current density 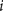 depends on factors such as temperature and the electrochemical characteristics of the catalyst. As shown in equation (4), the factor of the catalyst specific area and the exchange current density multiplication 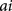 is determined by factors such as the structural feature of the electrode, including the reaction interface roughness and the mean radius of the catalyst particles, and is the most important factor for catalyst cost reduction, where 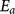 is the activation energy for the ORR; 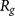 is the universal gas constant, 8.314 J/mol K; and 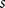 is the liquid water saturation. The ratio of 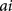 of ‘Layer 1’ and ‘Layer 2’ is given in equation (5).
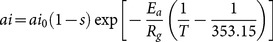
(4)

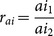
(5)

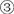
 A lumped variable 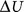 is defined in equation (6), in which 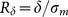 is the overall ionic resistance across the cathode electrode, and 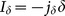 is the current density based on the transfer current density 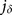 at the interface between the two electrodes.
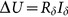
(6)

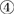
 The relative location of the interface between the two sub-layers is defined in equation (7), in which 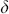 is the total thickness of the dual-layer electrodes.
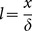
(7)

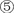
 The thickness ratio of the two sub-layers 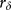 is defined in equation (8)
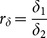
(8)

Considering the cathode electrode in one dimension (x direction), the two indices (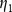 and 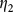) of the over-potential difference of ‘Layer 1’ and ‘Layer 2’ are given in equations (9) and (10), respectively, where 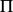, 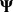 and 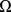 are defined in equations (11) to (13) [21].
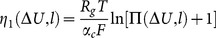
(9)

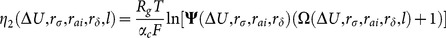
(10)

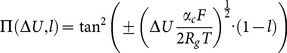
(11)

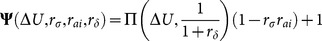
(12)

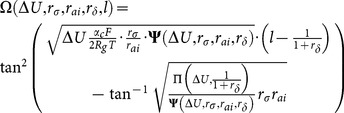
(13)


## Bat swarm algorithm with variable population

**Figure 2:**
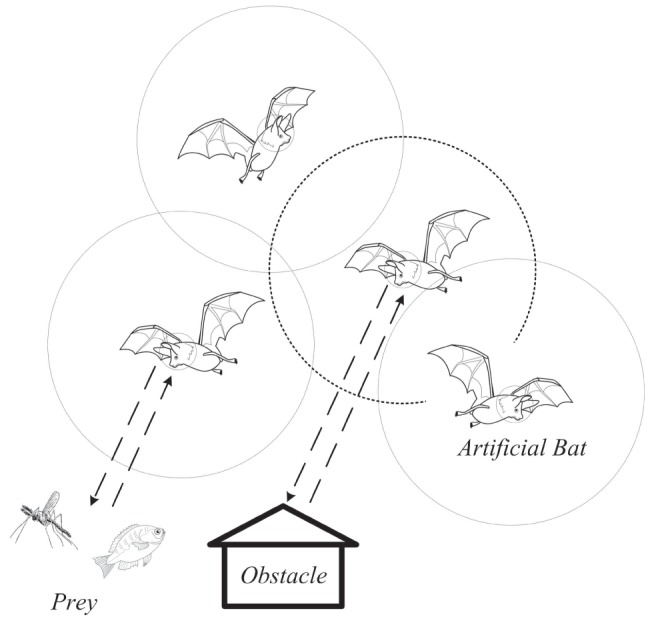
The behaviors of bat swarms.



Because the BAVP is inspired by the echolocation characteristics of bat swarms, it can be idealized to include the four following assumptions:

- 1 As shown in Fig. 2, all artificial bats (ABs) utilize the same echolocation mechanism to measure distance, and each AB individual 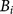 is able to detect the difference between prey (food) and obstacles.
- 2 Each individual 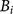 can generate ultrasounds to echolocate the prey and obstacles with a velocity of 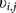 and a position of 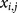 at time 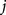, which are stated in Equations (15) and (14), respectively, where 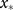 is the current global best position.
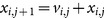
(14)

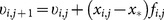
(15)
- 3 Each individual 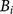 can adjust the frequency of the ultrasounds 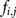 at time 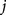 within a range of [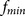, 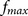], corresponding to a wavelength 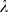 in the range of [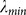, 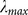] and a loudness 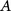 in the range of [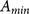, 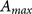], as given in Equation (16), where 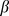 is a random vector of uniform distribution in the range of [0,1].
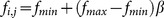
(16)
- 4 As shown in Equation (17), the population 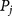 of ABs varies from time 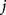 to another, which accelerates the optimization process, in which 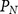 is the non-replaceable population and 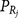 is the replaceable population at time 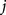.

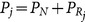
(17)


**Figure 3:**
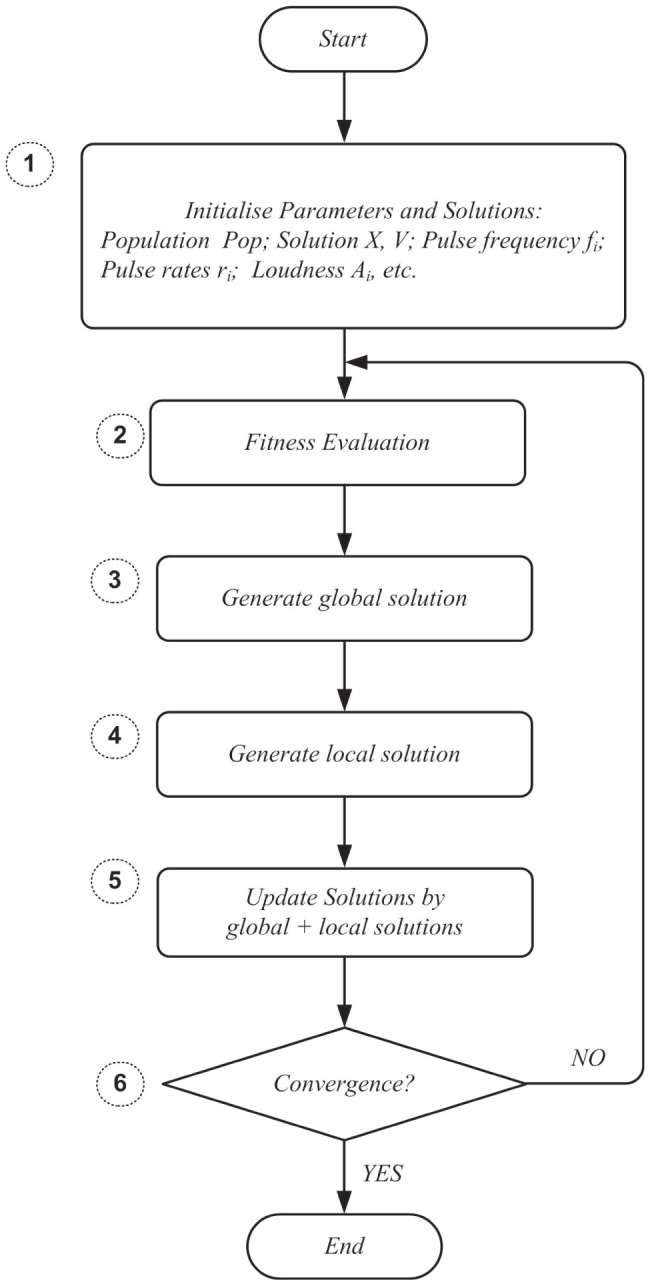
BAVP flowchar.

As shown in Fig. 3, the following six steps are included in the BAVP flow chart: step (1), initialization; step (2), fitness evaluation; step (3), global solution generation; step (4), local solution generation; step (5), update solutions by using the global and local solutions; and step (6), check termination condition of convergence.

- Step (1), start program and initialize parameters and solutions; all artificial bats are moving randomly.
- Step (2), evaluate fitness for each solution.
- Step (3), generate new global solutions 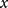, update velocities and adjust frequencies using equations (14) to (16).
- Step (4), generate new local solutions 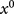 using Equation (18), where 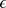 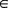 [−1,1] is a random-walk factor. As defined in Equation (19), 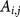 is the loudness of the bat 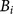 at time 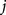, in which 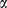 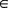 [0,1] is a reduction factor.
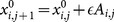
(18)

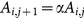
(19)
- Step (5), compare the local and global solutions and update solutions, as given in Equation (20).
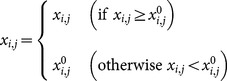
(20)
- Step (6), continue running the calculation until the terminal conditions have been satisfied.

## Computational intelligence-aided design

Computational intelligence (CI) is a set of nature-inspired approaches that provides numerous capabilities for solving complex problems. Compared to the traditional optimization methods, CI does not need to reformulate the problem to search a non-linear or non-differentiable space. Another advantage of CI is its flexibility in formulating the fitness function, which can be expressed as a function of the system output. This feature is particularly appealing if an explicit objective function is difficult to obtain.

**Figure 4:**
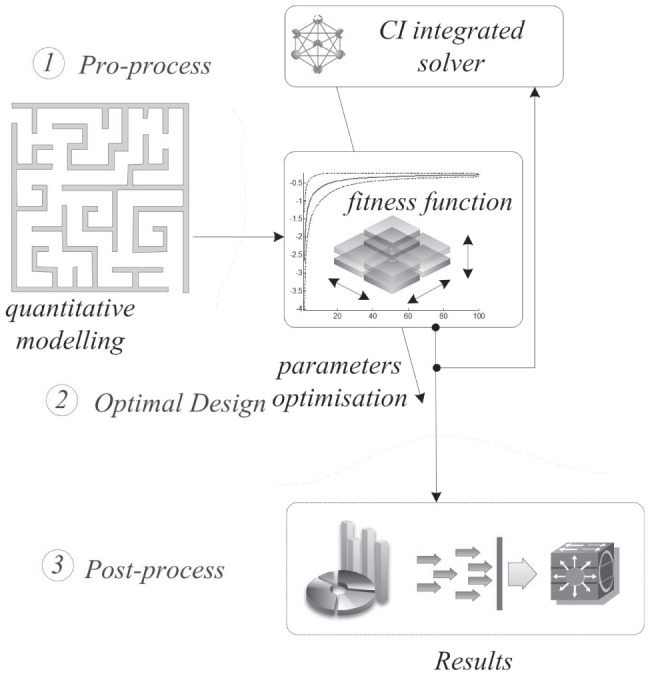
Framework of computational intelligence-aided design.

**Figure 5:**
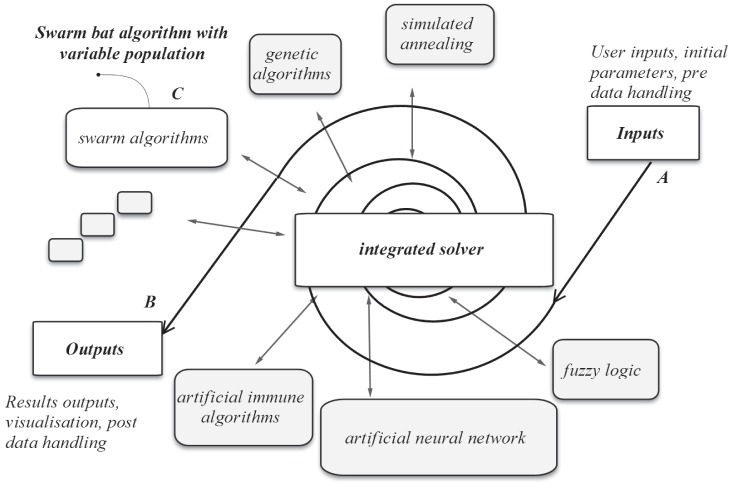
The conceptual framework of the computational intelligence integrated solver.

Fig. 4 illustrates the CIAD framework, and the entire optimization process can be summarized in the following three main steps:

- *Step 1*, pre-process. In this step, quantitative models under specific conditions are obtained for engineering applications.
- *Step 2*, optimal design. This step defines the fitness functions according to the design objectives.
- *Step 3*, post-process. This step produces the final results and completes the post-processing tasks. Specifically, this step reports the optimal solution, analyses and visualizes the results, and presents the recommendations to policy makers. The ‘CI integrated solver’(CIS) is employed to optimize the parameters for the fitness function, and the details of the CIS are given in Fig. 5.

As shown in Fig. 5, the conceptual framework of CIS consists of three parts: data input, the CI integrated solver and result output, as follows.

1. Part 1: Data Input (@point A). This part prepares the data input for the CI integrated solver. It collects, filters, stores, and pre-processes data originating from various sources, such as statistical yearbooks, research analyses, and government reports.
2. Part 2: CI Integrated Solver. In this part, a set of nature-inspired computational approaches are integrated into one solver to optimize complex real-world problems, which primarily involves one or more of the following methods: artificial neural networks, a genetic algorithm, fuzzy logic, simulated annealing, artificial immune algorithms, and swarm intelligence algorithms. In this paper, a BAVP algorithm (@point C) is embedded in this solver, and the details of this algorithm are discussed in Section 3.
3. Part 3: Result Output (@point B). This part reports the final results from Part 2. As shown in Fig. 4, the data-flow from Steps 4 to 3 is the input of the ‘CI integrated solver’ interconnected with point A, and the data-flow from Steps 3 to 2 is the output of the ‘CI integrated solver’ interconnected with point B.

## Optimization and Parameter Determination

To determine the optimal parameters for the over-potential difference 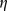, this section introduces two trend indices *mmAP* and *mmVAR* for evolutionary optimization, which are given in equations (21) and (22), respectively.

As stated in Equation (21), the index of *mmAP* is a moving average score of the mean value of vector 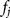, where 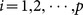, 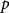 is the population of the data set, and *MEAN* 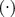 is the average function. The index of *mmVAR* is a moving average score of the VAR value of vector 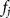, as given in Equation (22), where *VAR* 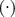 is the variance function. The two indices are employed to assess the short-term fluctuations by capturing the longer-term trend across the evolutionary process.
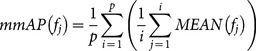
(21)

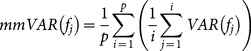
(22)


**Figure 6:**
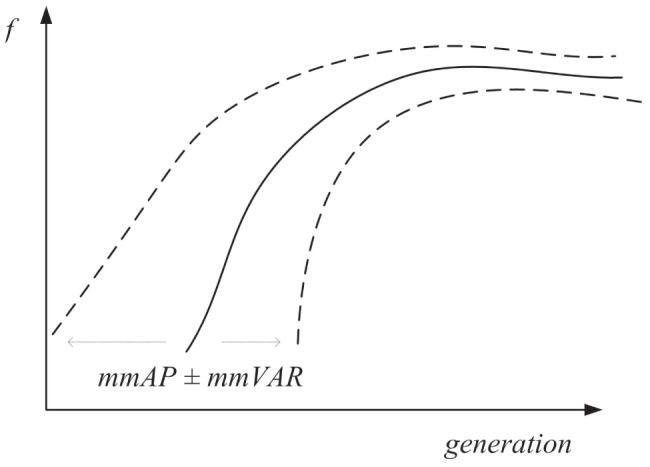
The diagram of mmAP 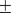
 **mmVAR over the full generations.**

In Fig. 6, the solid line represents the *mmAP* scores for each vector 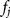 as given in Equation (21). The dashed lines are the *mmAP* 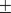 *mmVAR* for each vector 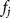 as given in equation (22), which defines the limits of evolutionary paths of the optimization process (generation versus fitness 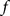) as the upper and lower boundaries.

The fitness function 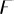 is in a reciprocal form of the over-potential difference function, as given in Equation (23). The fitness function is defined as the *mmAP* reciprocal function of the over-potential difference function 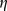, in which maximizing 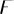 is a way to minimize 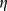, and the goal of this function is to determine the optimal combination of five parameters, 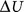, 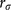, 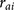, 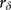 and 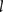, that simultaneously minimizes the objective of 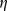. 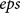 is the floating-point relative accuracy, which prevents singularity in the case where 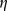 is approaching 0 and 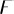 is approaching 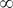.
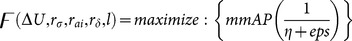
(23)


The over-potential difference function 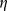 is given in Equation (24), where 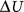 is the lumped variable, given in Equation (6); 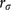 is the ratio of the ionic conductivity of the two sub-layers, given in Equation (3); 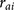 is the ratio of 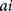, given in Equation (5); 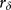 is the ratio of the thickness, given in Equation (8); and 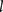 is the location factor, given in Equation (7).
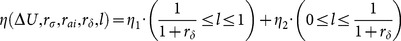
(24)


## Empirical Results and Discussion

Maximizing the fitness function 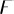 yields the minimum of 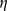, which is performed using the specially designed toolboxes 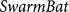 [49] and 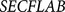 [50]. The computer specifications for the simulations are a 2.1 GHz Intel dual-core processor, Windows XP Professional v5.01 Build 2600 service pack 3, a 2.0 GB 800 MHz dual channel DDR2 SDRAM, and MATLAB R2008a.

The initial parameters are listed in Table 1, in which the max-generation number is 100, and it serves as the termination condition in each test. The test number is also 100. The frequency range is set to [20000,500000]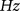. The reduction factor 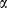 is 0.9. The population is 50, in which the non-replaceable 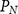 and replaceable population 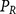 are 40 and 10, respectively. The random step is 0.01, and the ranges of 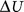, 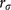, 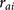, 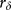, and 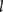 are [0,10], [0,10], [0,10], [0,2] and [0,3], respectively.

**Table 1:** Parameters of the Swarmbat optimization.

max generations		100
test number		100
frequency range		[20000,500000] 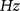
reduction factor	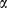	0.9
population		50
random step		0.01
lumped variable	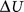	[0,0.1]V
ratio of ionic conductivity	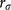	[0,2]
ratio of 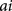	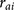	[0,2]
ratio of thickness	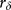	[0,4]
location factor	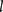	[0,1]

Table 2 presents the optimal combinations (MEAN
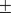
VAR) of 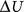, 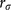, 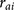, 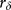 and 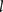, which indicates that the over-potential is non-uniform within the cathode and at particularly high values of the lumped parameter 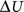 and is sensitive to the spatial variation 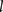.

**Table 2:** Optimal results of ‘MEAN
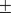
VAR’.

Parameters	Results
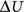	0.07754 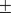 0.01034 V
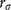	1.03191 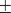 0.12681
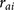	0.85241 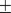 0.13736
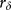	2.05281 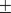 0.16181
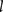	0.40709 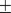 0.15818

**Figure 7:**
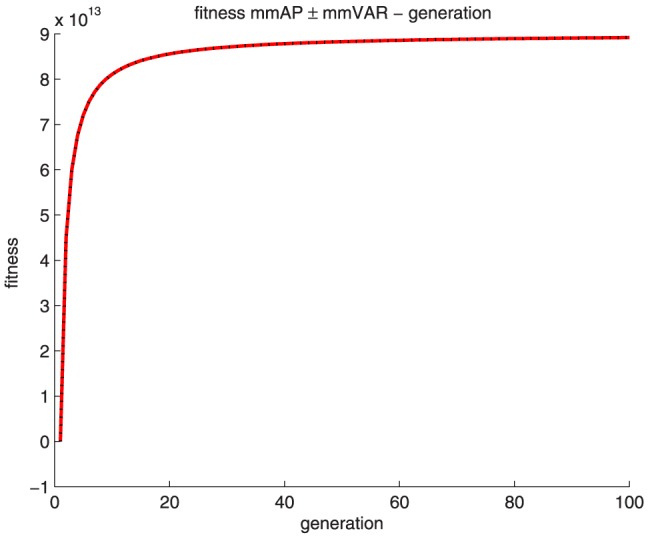
Fitness curves of mmAP 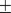
 **mmVAR over the simulation.**

Fig. 7 shows the *mmAP* curves with the upper and lower *mmVAR* boundaries, in which the *mmVAR* boundaries stick to the *mmAP* fitness curves and the fitness increases very quickly; it reaches a plateau from generations 1 to 60 (or so), and it remains steady from generation 60 to 100. Note that all lines converge in generation 100.

**Figure 8:**
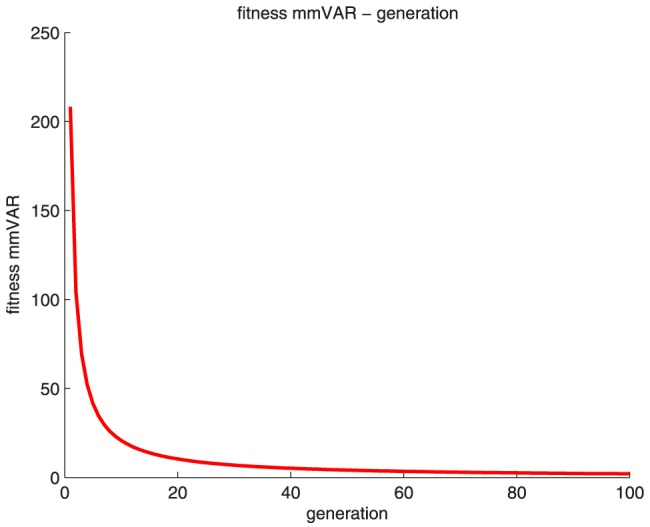
Fitness curves of mmVAR over the simulation.

Fig. 8 shows the fitness *mmVAR* over the entire simulation. The curves decline quickly within approximately 60 generations and finally reach 0 in generation 100. Figs. 7 and 8 indicate that the proposed optimization algorithm is efficient and accurate.

**Figure 9:**
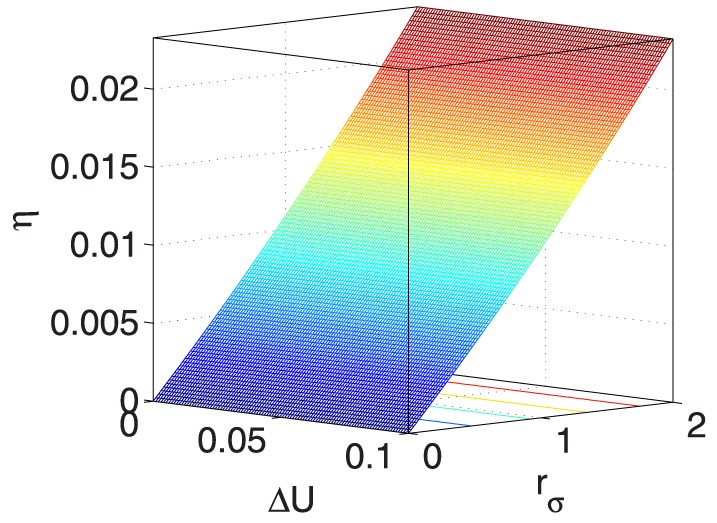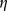 **vs.** 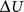 **and** 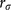 **.**

**Figure 15:**
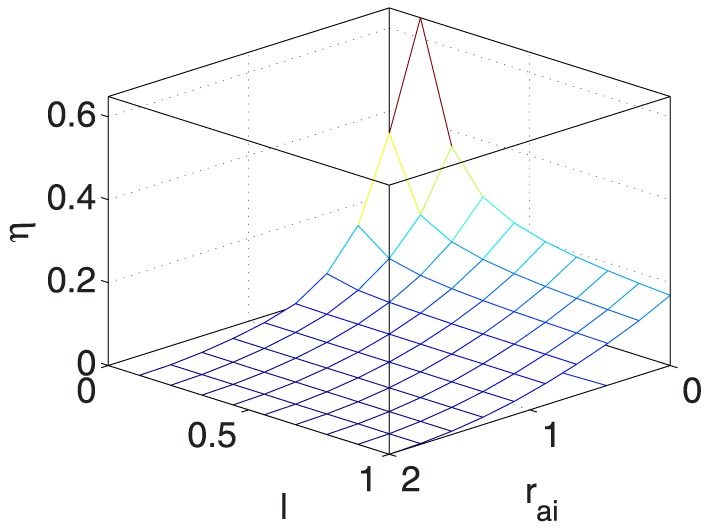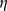 **vs.** 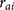 **and** 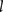 **.**

As also listed in Table 3, to demonstrate the impacts of the five coupled variables on 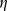, Figs. 9 and 15 provide seven ‘3D’ figures to evaluate these impacts.

**Table 3:** Impacts of five coupled variables on 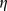 in 3D figures.

Figure No.	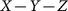 axis
Figure 9	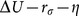
Figure 10	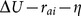
Figure 11	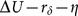
Figure 12	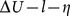
Figure 13	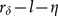
Figure 14	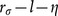
Figure 15	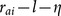

**Figure 10:**
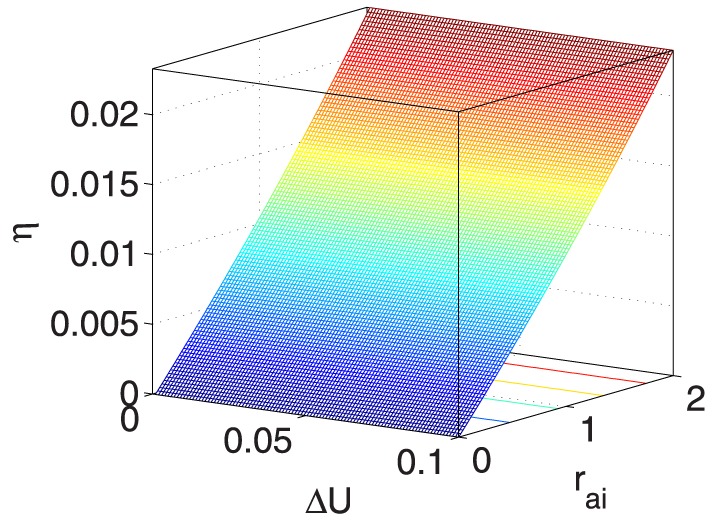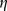 **vs.** 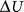 **and** 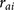 **.**

Specifically, Figs. 9 and 10 show that 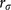 and 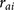 have similar positive effects on 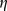, indicating that when 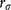 and 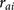 increase, 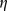 increases, and vice versa. Furthermore, when 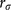 and 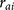 remain constant, 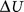 has limited effects on 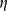.

**Figure 11:**
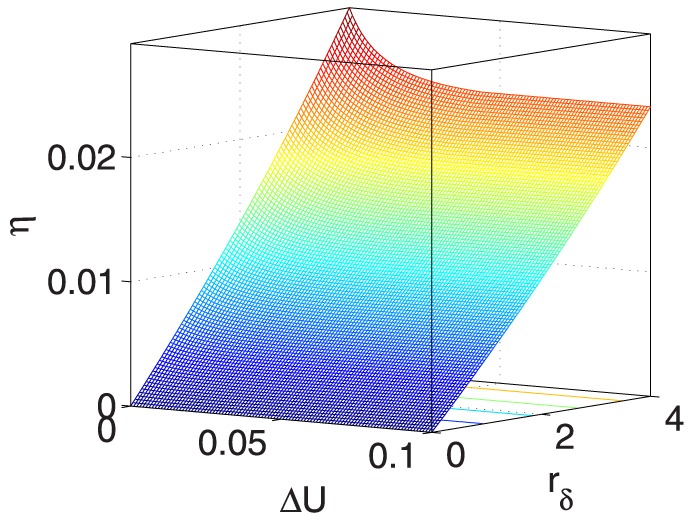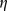 **vs.** 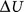 **and** 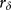 **.**

**Figure 12:**
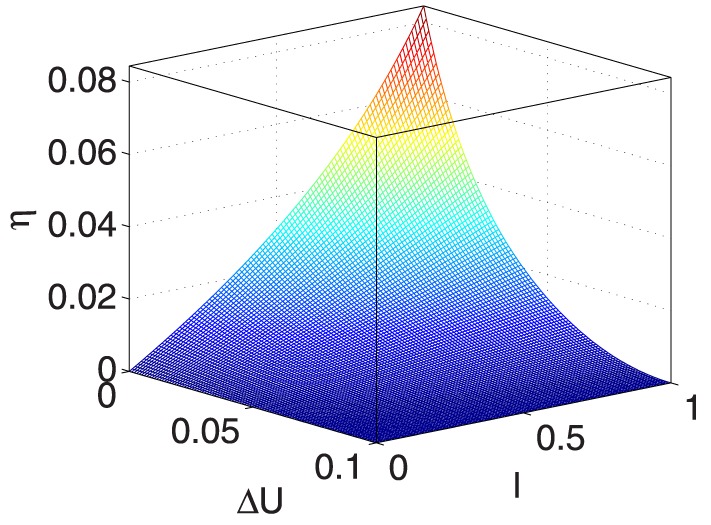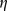 **vs.** 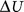 **and** 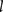 **.**

Fig. 11 shows that 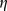 is sensitive to 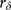 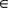 [3], [4] and 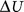 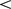 0.05. Fig. 12 indicates that 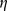 increases faster with 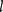 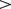 0.5 and 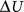 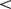 0.05 and that better 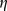 values are obtained with larger 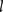 and smaller 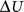.

**Figure 13:**
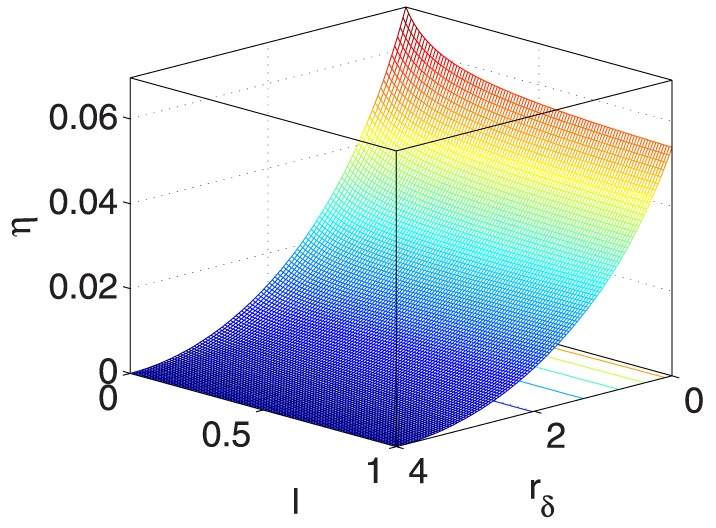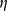 **vs.** 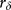 **and** 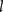 **.**

**Figure 14:**
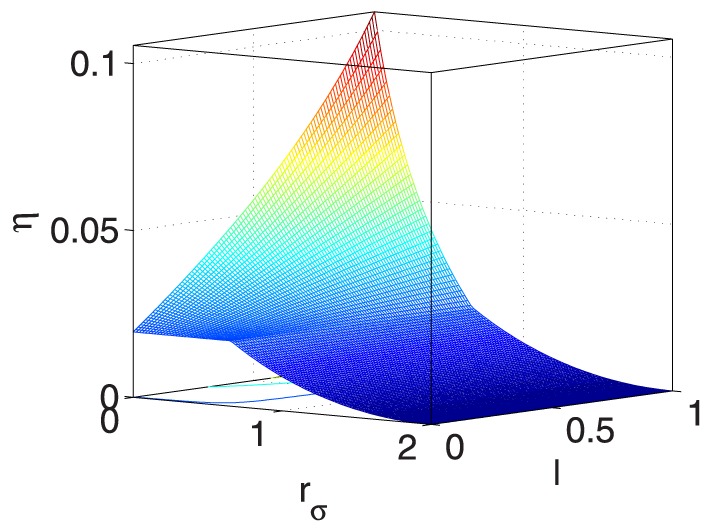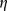 **vs.** 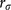 **and** 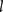 **.**

Fig. 13 shows that 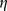 is sensitive to smaller 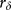 or 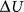. In Fig. 14, there is a plateau within 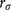 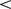 1 and 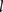 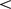 0.5; furthermore, 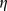 is sensitive to larger 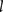 when 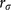 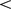 1. When 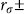 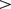 1 and increases, 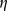 decreases within the full 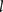 range.

Fig. 15 shows that 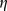 increases when both 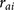 and 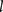 become smaller, which implies that 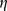 is unstable with small values of 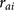 and 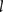.

## Conclusions and Future Works

In this study, an analytical model that incorporates five parameters is proposed to explore the transport and electrochemical phenomena in dual-layered cathode electrodes of polymer electrolyte fuel cells. These parameters include the lumped variable 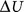, the ratio of the ionic conductivity of two sub-layers 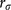, the 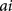 ratio of the two sub-layers 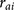, the ratio of the thickness 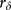 and the relative location factor 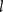. Moreover, a theoretical study on the spatial distribution of reaction rates across the electrode is presented.

The proposed model is utilized to define a design objective: determining the optimal combinations of the five parameters to minimize the over-potential difference 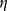. Based on the trend indices mmAP and mmVAR, a fitness function was constructed with the five variables as discussed above, which are optimized by the bat swarm algorithm with a variable population.

The numerical solutions obtained in this study were applied to optimize the electrode performance through a set of optimal dual-layer configurations, and the research findings are summarized in the following three points:

1. The proposed dual-layered cathode electrode model for the determination of the optimal parameters provides a strong argument for implementing the solutions to explore the impacts of each layer's properties on their performance.
2. Based on the developed dual-layered cathode electrode model, a bat swarm algorithm with a variable population is developed, which directly affects the determination of the optimal parameters due to its high efficiency and accuracy.
3. The proposed two trend indices *mmAP* and *mmVAR* were utilized to smooth out short-term fluctuations and highlight longer-term trends until the maximum generation fitness point was reached, which helps to measure the computational performance of the BAVP or to deploy other algorithms.

Our future research will focus on developing new types of CI algorithms, such as the swarm dolphin algorithm [51], the swarm wolf algorithm and their hybrid derivatives, to optimize further geometrical parameters and optimal combinations for improving the efficiency of polymer electrolyte fuel cells with multiple-layer configurations. To achieve a ‘state-of-practice’ design framework for the fuel cell, further experimental research is needed to establish an advanced model for chemical-dynamical coupled behavior and the potentials of fuel cell commercialization.
